# Supplementary material for: Effects of intraoperative hemodynamic management on postoperative acute kidney injury in liver transplantation: An observational cohort study
Source: PLoS One. 2020 Aug 18;15(8):e0237503. doi: 10.1371/journal.pone.0237503 (PMC7446917; doi:10.1371/journal.pone.0237503)
Supplement: S1 Appendix — (PDF) [file pone.0237503.s001.pdf]

SUPPLEMENTARY MATERIAL

Effects of intraoperative hemodynamic management on postoperative acute kidney injury after liver transplantation: an observational cohort study.

Appendix 1. Donor consent form in the Province of Quebec

AH-224A DT9118 (rev. 2011-02)

CONSENT TO REMOVE ORGANS AND TISSUES

- **Carefully read the instructions before completing the form. Once you have read the instructions, detach this section.**
- **Use a ballpoint pen only, and press firmly.**
- **Detach section 1 before completing the form. Then, complete section 2 without detaching the coupon at the bottom of the page.**

On page 1 of the form:

- In the box at the top right: Use the deceased's hospital card to identify the potential donor.
- For statistical purposes only, if the request was made but the donation was turned down, check the box at the top right under the identification of the deceased.
- Death*: The first paragraph in the box at the bottom of the page provides a specific definition of the word "death" in the context of organ donations.
- Quality of the signatory**: The second paragraph in the box at the bottom of the page indicates, **by order of priority**, the persons authorized by the Québec Civil Code to sign the consent form. De facto spouses are now considered equal to all other types of spouse and, as such, have the same authority to sign the consent form.
- Unrestricted consent: If family members have formulated no restrictions with regard to the organs and tissues that can be collected, all they have to do is place their initials after the sentence "I consent to the removal of organs and tissues without restrictions."
- Restricted consent: If family members have formulated restrictions with regard to the organs and tissues that can be collected, they must **place their initials next to each organ and tissue that can be removed**.

On page 2 of the form:

- Name of the deceased and file number: Since there are two detachable sheets, it is important to properly identify both pages of the form with the name of the deceased.
- The paragraphs that follow provide information to family members of the potential donor concerning the collection of medico-social information on the deceased, the collection of blood samples to test for infectious agents such as HIV, hepatitis, and the transfer of the potential donor, as required.
- Point 2 addresses consent for research and education. It also applies to studies carried out by Héma-Québec to improve the quality of the human tissue supply. Certain criteria have been established in the interest of ensuring the safety of recipients. When potential donors do not meet these criteria, some or all of their organs and tissues may not qualify for transplantation or grafts. In these cases, organs and tissues may still be donated for research, education, or studies carried out by Héma-Québec. Also, certain tissues which are never used for grafts, like the spinal column and the adrenal glands, can also be donated exclusively for research, education, and studies. Family members of the deceased must indicate their choice by placing their initials next to their choice (YES or NO). They may also indicate restrictions, if any.
- The last sentences before the signature stipulate that the signatory or signatories have read this consent form, that they were able to ask questions, that the required explanations were provided, and that all the information in the form was entered before they signed it.
- The person who obtained the consent must sign it, print his or her name on it, and indicate whether the consent was obtained by phone or not.

DT9118

CONSENT TO REMOVE ORGANS AND TISSUES

☐ Family approached, does not consent to the donation of organs and tissues

1) Consent to the donation of organs and tissues

Once the death* has been certified,

I, ______________________ (Name of signatory of the consent), ______________________ (Quality of the signatory** (compulsory))

of ______________________ (Name of the deceased), authorize the various teams to remove the organs and tissues for transplantation or graft purposes.

**I consent to the removal of organs and tissues <u>without</u> restrictions.**	**Initial if you agree.**
**I consent to the removal <u>with</u> restrictions.**<br>Place your initials beside the organs and tissues **THAT CAN BE REMOVED** for transplantation or graft purposes.	
**Organs**	**Tissues**
Heart .... ______	Heart (for valves) .... ______
Lungs .... ______	Pericardium .... ______
Liver .... ______	Blood vessels (arteries and veins) .... ______
Pancreas .... ______	Pancreas (for islets of Langerhans) .... ______
Intestines .... ______	Eyes .... ______
Kidneys .... ______	Bones .... ______
	Skin .... ______
	Tendons, ligaments, meniscus, and fascia ... ______
**Other (specify):**	

* In the context of organ donations, the ***death*** of an individual means ***neurological death***, which is defined as the irreversible loss of the state of consciousness and of all functions of the brainstem, including the ability to breathe despite the fact that the heart continues to beat and that spinal reflexes may persist.

** The signatory of this form must be authorized to sign it in accordance with sections 14, 15, and 44 of the Civil Code of Québec. For a person of full age, this is, ***by order of priority***, the mandatary, the tutor, the curator, the married, civil union, or de facto spouse, or a person who shows a special interest in the deceased person. For a minor, this is the person having parental authority or the tutor.

DT9118

Name of the deceased	
File No.	

I authorize the institution's director of professional services to transmit to the parties designated by the Minister all necessary medical information on the deceased to determine his or her eligibility to donate organs and tissues (*Act respecting health services and social services*, sec. 204.1).

I also authorize the intervening parties designated by the Minister to consult all the medical files of the deceased in order to determine his or her eligibility to donate organs and tissues.

I understand that samples to test for infectious agents including, among others, HIV, hepatitis B and C, and syphilis, will be taken and that blood samples may be kept for future analysis. I also understand that the information related to these tests will be kept confidential according to the legislation in force. The results will be transmitted to the public health authorities in the event a reportable disease is detected.

In the case of an organ donation, if the results must be transmitted to the family or close friend of the donor, please send the results to:

First and last name of the family physician or of the family member(s) or friend (optional): ______________________

I understand that the parties designated by the Minister may contact me, if needed, to determine the medico-social history of the deceased.

I authorize, if necessary, the transfer of the deceased to an organ and tissue removal centre.

2) Consent to the removal of organs or tissues for education and research

If the organs or tissues cannot be used for the purposes of transplantation or grafts, I consent to their removal and anonymous use for:

- Research projects preapproved by a research ethics committee
- Education
- Studies carried out by Héma-Québec to improve the quality of the human tissue supply

Place your initials
next to your choice. YES ________ NO ________ Restriction(s): ______________________

I certify that

- **I have read (alone or with assistance) and understand this consent form.**
- **I was able to ask questions and explanations regarding this document were provided to me.**
- **All the information on this form was entered on it before I signed it.**

Signature ______ Name (capital letters) ______ Date ______ Time ______

Address of signatory ______ Postal Code ______ Area Code ______ Phone No. ______

I have explained this consent form and I have answered all the signatory's questions.

Signature of the person who obtained the consent ______ Name of the person who obtained the consent ______

Consent by phone: ☐ Yes ☐ No
