# Supplementary material for: Effects of intraoperative hemodynamic management on postoperative acute kidney injury in liver transplantation: An observational cohort study
Source: PLoS One. 2020 Aug 18;15(8):e0237503. doi: 10.1371/journal.pone.0237503 (PMC7446917; doi:10.1371/journal.pone.0237503)
Supplement: S2 Appendix — (DOCX) [file pone.0237503.s002.docx]

**SUPPLEMENTARY MATERIAL**

***Effects of intraoperative hemodynamic management on postoperative acute kidney injury after liver transplantation: an observational cohort study.***

**Appendix 2. Intraoperative management, selection of confounders and outcomes and conceptual framework of the research question**

**Intraoperative management**

At our institution, anesthesiologists lowered central venous pressure (CVP) by limiting fluid administration and by performing a phlebotomy in selected recipients (patients with a hemoglobin concentration > 85 g/L and a normal renal function) to reduce portal pressure and blood loss. Our phlebotomy consists of a removal of 7-10 ml/kg of blood before the dissection phase, without fluid replacement, that is transfused back at graft reperfusion. [1,2] We transfused hemostatic blood products only when overt bleeding was observed and based on central laboratory conventional coagulation tests: fresh frozen plasma for an INR > 1.5, platelets pool or apheresis for a platelet count ≤ 30 × 10^9^/L and cryoprecipitate pool (10 units < 2017 and 5 units after) for a fibrinogen concentration ≤ 2 g/L. We administered a tranexamic acid infusion routinely (bolus of 2 g + 1 g/h up to graft reperfusion). We used a cell saver device in all patients and transfused red blood cells when hemoglobin concentration fell below 70 g/L or during active bleeding. We used transoesophageal echocardiography for selected high-risk cases or in case of severe intraoperative hemodynamic instability. We used either norepinephrine, phenylephrine or vasopressin infusions to maintain blood pressure based on anesthesiologist’s preference. Cardiac output was not measured routinely.

**Confounding**

This study objective was to better define the role of vasopressors either as an independent exposure variable, as a fluid balance effect measure modifier or as a confounder (figure S1).[3] Other exposures of interest were the intraoperative fluid balance and the performance of a phlebotomy. Many potential confounders of the association between the aforementioned exposures and our outcomes were identified: intraoperative hemodynamic instability, preoperative renal failure and anemia, severity of liver disease, intraoperative signs of hypervolemia, central venous pressure (CVP), heart failure and severe coagulopathy. [4] Hemodynamic instability itself was not measured in this study and might be an important confounder based on both physiological reasoning and published literature.[5-10] Severity of liver failure has been already shown to be associated with worse postoperative outcomes and an increased risk of acute renal failure.[11] Severity of liver failure was evaluated by the MELD-NA and by the presence of acute liver failure as the transplantation indication. We hypothesized that coagulopathy was captured by the MELD-Na, but since our primary outcome was acute kidney injury (AKI) and it has been highly reported by anesthesiologists to influence their practice, we decided to add the pre-operative creatinine value as a potential independent confounder, as well as preoperative diabetes status. Preoperative hemoglobin concentration and the intraoperative use of phlebotomy has also been associated with the risk of bleeding and transfusions.[12] Baseline CVP might be a surrogate marker of both the patient’s volume status and heart function status, has been used to control fluid infusion in some studies [13] and also, by itself, affects the fluid strategy used intraoperatively in our center. Moreover, it has been recently associated with intraoperative bleeding and transfusions in a local 800 patient cohort. [14] Post-reperfusion syndrome is a vasoplegic shock after transplantation that is associated with renal failure and could by a significant confounder. We added the cold ischemia time, because it is associated with the post-reperfusion syndrome, and doses of vasopressors as a surrogate for this syndrome. [4,15] We added the length of vena cava clamping as well as the technique used, because this surgical intervention might increase the need for fluid resuscitation to maintain hemodynamic stability and could be associated with AKI. Finally, we added intraoperative exposure to starch, since this intervention is now known to be associated with AKI. [16]

**Figure S1. Conceptual framework for causal association of vasopressors and fluid management**

* *An intraoperative restrictive fluid management approach would be associated with higher vasopressor doses. Hemodynamic instability might be associated with both greater fluid administration and higher vasopressor doses. We explored both the effect measure modifying effect and the confounding effect of vasopressor on the fluid balance/complications association and supposed no mediating effect of vasopressors in our models.* [3]

**Outcomes**

We chose AKI as a primary outcome because of its high incidence following liver transplantation (13-71%) and its association with intraoperative events, the burden of postoperative care and mortality. [6,10,11,17-20] Postoperative AKI have mostly been evaluated after 7 days or less both in liver transplant recipients [6,10,11,18,21,22] and in other surgical populations. [23] The CLIF consortium, an European research collaborative organization focused on chronic liver failure, recently published guidelines on the management of critically ill cirrhosis patient in which they recommended the use of the KDIGO-AKI criteria to evaluate acute renal failure in these patients.[24] We chose the 48-hour timeline as the primary outcome because we presumed it would have a better association with the intraoperative period, that it might be less influenced by the nephrotoxicity of immunosuppressive drugs than the 7-day outcome and the better feasibility to measure urine output adequately at 48 hours then later. However, we also evaluated AKI at 7 days. We also looked at more intermediate outcomes, such as time to first extubation, time to intensive care unit (ICU) discharge as well as survival up to 1 year.

**References for supplementary material**

***Note: reference numbers are different from the ones in the main manuscript***

1. Massicotte L, Carrier FM, Denault AY, Karakiewicz P, Hevesi Z, McCormack M, et al. Development of a Predictive Model for Blood Transfusions and Bleeding During Liver Transplantation: An Observational Cohort Study. Journal of Cardiothoracic and Vascular Anesthesia. 2018;32: 1722–1730. doi:10.1053/j.jvca.2017.10.011

2. Massicotte L, Perrault M-A, Denault AY, Klinck JR, Beaulieu D, Roy J-D, et al. Effects of Phlebotomy and Phenylephrine Infusion on Portal Venous Pressure and Systemic Hemodynamics During Liver Transplantation. Transplantation. 2010;89: 920–927. doi:10.1097/TP.0b013e3181d7c40c

3. Greenland S. Quantifying biases in causal models: classical confounding vs collider-stratification bias. Epidemiology. 2003;14: 300–306.

4. Carrier FM, Chassé M, Sylvestre M-P, Girard M, Legendre-Courville L, Massicotte L, et al. Effects of intraoperative fluid balance during liver transplantation on postoperative acute kidney injury: an observational cohort study. Transplantation. 2019. doi:10.1097/TP.0000000000002998

5. Prasad V, Guerrisi M, Dauri M, Coniglione F, Tisone G, De Carolis E, et al. Prediction of postoperative outcomes using intraoperative hemodynamic monitoring data. Sci Rep. Nature Publishing Group; 2017;7: 16376. doi:10.1038/s41598-017-16233-4

6. Sirivatanauksorn Y, Parakonthun T, Premasathian N, Limsrichamrern S, Mahawithitwong P, Kositamongkol P, et al. Renal dysfunction after orthotopic liver transplantation. Transplantation Proceedings. 2014;46: 818–821. doi:10.1016/j.transproceed.2013.11.124

7. De Maria S, Nürnberg J, Lin HM, Contreras-Saldivar AG, Levin M, Flax K, et al. Association of intraoperative blood pressure instability with adverse outcomes after liver transplantation. Minerva Anestesiol. 2013;79: 604–616.

8. Cabezuelo JB, Ramírez P, RIOS A, Acosta F, Torres D, Sansano T, et al. Risk factors of acute renal failure after liver transplantation. Kidney Inter, Suppl. 2006;69: 1073–1080. doi:10.1038/sj.ki.5000216

9. Mizota T, Hamada M, Matsukawa S, Seo H, Tanaka T, Segawa H. Relationship Between Intraoperative Hypotension and Acute Kidney Injury After Living Donor Liver Transplantation_ A Retrospective Analysis. Journal of Cardiothoracic and Vascular Anesthesia. Elsevier Inc; 2017;31: 582–589. doi:10.1053/j.jvca.2016.12.002

10. Thongprayoon C, Kaewput W, Thamcharoen N, Bathini T, Watthanasuntorn K, Lertjitbanjong P, et al. Incidence and Impact of Acute Kidney Injury after Liver Transplantation: A Meta-Analysis. JCM. 2019;8: 372–25. doi:10.3390/jcm8030372

11. Hilmi IA, Damian D, Al-Khafaji A, Planinsic R, Boucek C, Sakai T, et al. Acute kidney injury following orthotopic liver transplantation: incidence, risk factors, and effects on patient and graft outcomes. Hemmings HC, editor. British Journal of Anaesthesia. 2015;114: 919–926. doi:10.1093/bja/aeu556

12. Massicotte L, Capitanio U, Beaulieu D, Roy J-D, Roy A, Karakiewicz PI. Independent Validation of a Model Predicting the Need for Packed Red Blood Cell Transfusion at Liver Transplantation. Transplantation. 2009;88: 386–391. doi:10.1097/TP.0b013e3181aed477

13. Wang B, He H-K, Cheng B, Wei K, Min S. Effect of low central venous pressure on postoperative pulmonary complications in patients undergoing liver transplantation. Surg Today. 2012;43: 777–781. doi:10.1007/s00595-012-0419-y

14. Massicotte L, Carrier FM, Karakiewicz P, Hevesi Z, Thibeault L, Nozza A, et al. Impact of MELD Score-Based Organ Allocation on Mortality, Bleeding, and Transfusion in Liver Transplantation: A Before-and-After Observational Cohort Study. Journal of Cardiothoracic and Vascular Anesthesia. 2019;33: 2719–2725. doi:10.1053/j.jvca.2019.03.008

15. Paugam-Burtz C, Kavafyan J, Merckx P, Dahmani S, Sommacale D, Ramsay M, et al. Postreperfusion syndrome during liver transplantation for cirrhosis: Outcome and predictors. Liver Transpl. 2009;15: 522–529. doi:10.1002/lt.21730

16. Myburgh JA, Finfer S, Bellomo R, Billot L, Cass A, Gattas D, et al. Hydroxyethyl starch or saline for fluid resuscitation in intensive care. N Engl J Med. 2012;367: 1901–1911. doi:10.1056/NEJMoa1209759

17. Parikh A, Washburn KW, Matsuoka L, Pandit U, Kim JE, Almeda J, et al. A multicenter study of 30 days complications after deceased donor liver transplantation in the model for end-stage liver disease score era. Liver Transpl. 2015;21: 1160–1168. doi:10.1002/lt.24181

18. Wiesen P, Massion PB, Joris J, Detry O, Damas P. Incidence and risk factors for early renal dysfunction after liver transplantation. WJT. 2016;6: 220–232. doi:10.5500/wjt.v6.i1.220

19. Pereira AA, Bhattacharya R, Carithers R, Reyes J, Perkins J. Clinical factors predicting readmission after orthotopic liver transplantation. Liver Transpl. 2012;18: 1037–1045. doi:10.1002/lt.23475

20. Ojo AO, Held PJ, Port FK, Wolfe RA, Leichtman AB, Young EW, et al. Chronic renal failure after transplantation of a nonrenal organ. N Engl J Med. 2003;349: 931–940. doi:10.1056/NEJMoa021744

21. Smoter P, Nyckowski P, Grat M, Patkowski W, Zieniewicz K, Wronka K, et al. Risk factors of acute renal failure after orthotopic liver transplantation: single-center experience. Transplantation Proceedings. 2014;46: 2786–2789. doi:10.1016/j.transproceed.2014.09.044

22. Massicotte L, Lenis S, Thibeault L, Sassine M-P, Seal RF, Roy A. Effect of low central venous pressure and phlebotomy on blood product transfusion requirements during liver transplantations. Liver Transpl. Wiley Subscription Services, Inc., A Wiley Company; 2006;12: 117–123. doi:10.1002/lt.20559

23. Pearse RM, Harrison DA, MacDonald N, Gillies MA, Blunt M, Ackland G, et al. Effect of a Perioperative, Cardiac Output–Guided Hemodynamic Therapy Algorithm on Outcomes Following Major Gastrointestinal Surgery. JAMA. 2014;311: 2181. doi:10.1001/jama.2014.5305

24. Nadim MK, Durand F, Kellum JA, Levitsky J, O’Leary JG, Karvellas CJ, et al. Management of the critically ill patient with cirrhosis: A multidisciplinary perspective. Journal of Hepatology. European Association for the Study of the Liver; 2016;64: 717–735. doi:10.1016/j.jhep.2015.10.019
