# Supplementary material for: Effects of intraoperative hemodynamic management on postoperative acute kidney injury in liver transplantation: An observational cohort study
Source: PLoS One. 2020 Aug 18;15(8):e0237503. doi: 10.1371/journal.pone.0237503 (PMC7446917; doi:10.1371/journal.pone.0237503)
Supplement: S3 Appendix — (DOCX) [file pone.0237503.s003.docx]

**SUPPLEMENTARY MATERIAL**

***Effects of intraoperative hemodynamic management on postoperative acute kidney injury after liver transplantation: an observational cohort study.***

**Appendix 3. Supplementary tables and figures**

**Table S1. Supplemental cohort baseline characteristics**

| **Variables** | **N = 532** |
| --- | --- |
| Demographics and laboratory values | |
| Age | 52 ± 11 |
| Sex (male) | 358 (67.3 %) |
| Hemoglobin level (g/L) | 106 ± 24 |
| Bilirubin level (umol/L) | 67 [32, 164] |
| Creatinine level (umol/L) | 77 [62, 106] |
| INR | 1.6 [1.3, 2.0) |
| Sodium (mmol/L) | 136 ± 5 |
| MELD | 20.5 ± 8.5 |
| Comorbidities | |
| Diabetes | 99 (18.6 %) |
| COPD/asthma | 30 (5.6 %) |
| Coronary artery disease | 18 (3.4 %) |
| Smoker status | 20 (3.8 %) |
| Preoperative liver disease diagnoses | |
| Viral cirrhosis | 104 (19.6 %) |
| Alcoholic cirrhosis | 98 (18.4 %) |
| NASH cirrhosis | 59 (11.1 %) |
| Mixed and other cirrhosis | 45 (8.5 %) |
| Autoimmune disease cirrhosis | 117 (22.2 %) |
| Primary biliary cholangitis | 47 (8.8 %) |
| Sclerosing cholangitis | 43 (8.1 %) |
| Autoimmune chronic hepatitis | 18 (3.4 %) |
| Mixed autoimmune disease | 9 (1.7%) |
| Primary liver cancer | 5 (0.9 %) |
| Other chronic liver diseases | 28 (5.3 %) |
| Acute liver failure | 20 (3.8 %) |
| Retransplantation | 56 (10.5%) |
| Graft cholangiopathy | 29 (5.5 %) |
| Graft thrombosis | 12 (2.3 %) |
| Graft primary dysfunction^*^ | 5 (0.9 %) |
| Graft recurrent disease | 6 (1.1 %) |
| Graft chronic rejection | 4 (0.8 %) |
| Hepatocellular carcinoma^**^ | 135 (25.4 %) |
| Hepatopulmonary syndrome^**^ | 9 (1.7 %) |

*This table is similar to one included in our previous publication (Carrier FM et al. Transplantation 2020).*

*Results are expressed as means ± standard deviation or as medians with [first and third quartiles].*

*^*^ Were considered as acute liver failure for the analyses*

*^**^ Not exclusive categories*

*Abbreviations: NASH = non-alcoholic steatohepatitis, INR = international normalized ratio, MELD = Model for End-stage Liver Disease, COPD = chronic obstructive pulmonary disease*

**Table S2. Important perioperative variables by vasopressor groups with statistical tests**

|  | **No vasopressors (n=283)** | **Vasopressors (n=241)** | **p-value^*^** |
| --- | --- | --- | --- |
| **Baseline characteristics** | | | |
| Age | 51 (12) | 54 (11) | < 0.001 |
| Sex (male) | 195 (69%) | 158 (66%) | 0.5 |
| Hemoglobin level (g/L) | 109 (25) | 102 (23) | 0.001 |
| Bilirubin level (umol/L) | 53 [25, 157] | 81 [40, 166] | 0.001 |
| Creatinine level (umol/L) | 74 [59, 98] | 85 [66, 115] | < 0.001 |
| INR | 1.5 [1.2, 1.9] | 1.6 [1.3, 2.1] | 0.06 |
| Sodium (mmol/L) | 136 (5) | 135 (6) | 0.04 |
| MELD | 19 (9) | 22 (8) | < 0.001 |
| Acute liver failure | 15 (5.3%) | 8 (3.3%) | 0.4 |
| Retransplantation | 31 (11%) | 24 (10%) | 0.8 |
| **Donor characteristics** | | | |
| Age | 51 (17) | 53 (16) | 0.08 |
| Sex (male) | 158 (56%) | 133 (55%) | 0.6 |
| CIT (hours) | 7.4 (2.2) | 7.4 (2.5) | 0.8 |
| Type of donation:   - Donation after NDD - DCD - Living | 280 (99%)  0 (0%)  3 (1%) | 233 (97%)  2 (1%)  6 (2%) | 0.3 |
| Cause of death (excludes living donation)   - Anoxia/hanging - Hemorrhagic stroke - Ischemic stroke - Subarachnoid hemorrhage - Traumatic brain injury - Other - Not reported cause of NDD | 46 (16%)  40 (14%)  97 (34%)  18 (6%)  68 (24%)  7 (2%)  4 (1%) | 46 (19%)  38 (16%)  87 (36%)  17 (7%)  40 (17%)  4 (2%)  3 (1%) | 0.5 |
| **Surgical variables** | | | |
| Vena cava clamping time (minutes) | 40 (17) | 43 (19) | 0.1 |
| Length of surgery (minutes) | 230 [200, 266] | 240 [210, 300] | < 0.001 |
| Piggyback caval anastomosis | 9 (3.2%) | 14 (5.8%) | 0.2 |
| **Anesthesiologic variables** | | | |
| Baseline CVP (mmHg) | 14.2 (5.4) | 14.5 (5.0) | 0.5 |
| Phlebotomy (exposed) | 179 (63.2%) | 115 (47.7%) | < 0.001 |
| Ascites (L)^+^ | 0.5 [0, 4.0] | 2.0 [0.5, 6.0] | < 0.001 |
| Intraoperative urine output (L) | 0.35 [0.22, 0.54] | 0.35 [0.20, 0.50] | 0.3 |
| Intraoperative bleeding (L) | 0.8 [0.5, 1.3] | 1.4 [0.8, 2.5] | < 0.001 |
| Crystalloid (L) | 3.8 [3.0, 4.5] | 4.0 [3.0, 5.0] | 0.004 |
| Colloid (L) | 0.5 [0, 0.5] | 0.5 [0, 1.0] | 0.009 |
| Cellsaver output (L) | 0.20 [0.10, 0.35] | 0.32 [0.18, 0.51] | < 0.001 |
| Intraoperative RBC transfusions (%) | 45 (15.9%) | 94 (39.0%) | < 0.001 |
| **Main exposure** | | | |
| Fluid balance (L)^++^ | 1.1 (3.3) | 0.2 (3.9) | 0.02^**^ |

*Results are reported as number of observed cases (proportion in %), as means (SD) or as medians [quartile 1, quartile 3].*

*N.B. 8 missing values for vasopressors. Missing values < 5 per group are not reported.*

*^*^All p values come from hypothesis tests conducted by Welch t-tests for normally distributed variables and Mann-Whitney U tests for non-normally distributed variables. These tests did not take into account correlation between data from the same patients and might be too liberal in rejecting the null hypothesis; they should be interpreted as exploratory tests only.*

*^**^ Means difference for fluid balance had a p value = 0.015 when fitted in a GEE linear model that took correlation into account.*

*^+^ 55 (19.4%) and 42 (17.4%) missing values respectively.*

*^++^ 58 (20.5%) and 44 (18.3%) missing values respectively.*

**Table S3. Time to first extubation results from multivariable analyses**

| **Variable** | **HR (n=521)** |
| --- | --- |
| Any vasopressor | 0.64 [0.50, 0.81]^*^ |
| Fluid balance (L) | 0.99 [0.95, 1.04] |
| Intraoperative phlebotomy | 1.04 [0.81, 1.35] |
| Sex (male) | 1.32 [1.01, 1.73]^*^ |
| Age | 1.01 [0.99, 1.02] |
| Retransplantation | 0.97 [0.61, 1.56] |
| ALF | 0.45 [0.21, 0.97]^*^ |
| MELD | 0.98 [0.96, 0.99]^*^ |
| Diabetes | 1.33 [1.01, 1.76] |
| CVP | 0.99 [0.97, 1.01] |
| CIT | 1.02 [0.97, 1.08] |
| Vena cava clamping | 0.99 [0.98, 0.99]^*^ |
| Piggyback | 0.84 [0.42, 1.69] |
| Preoperative creatinine | 1.00 [0.99, 1.00] |

*^*^ Significant at alpha level = 0.05.*

*A HR > 1 increases the risk of extubation. Results are expressed with 95% confidence intervals.*

*This model was a multivariable model stratified for hemoglobin and exposure to starch.*

*Interaction between vasopressor and fluid balance was not significant.*

*Abbreviations: HR = hazard ratio, ALF = acute liver failure, MELD = Model for End-stage Liver Disease, CVP = central venous pressure, CIT = cold ischemia time*

**Table S4. Time to ICU discharge results from multivariable analyses**

| **Variable** | **HR (n=525)** |
| --- | --- |
| Any vasopressor | 0.91 [0.76, 1.09] |
| Fluid balance (L) | Non-linear effect^*^ |
| Intraoperative phlebotomy | 1.17 [0.96, 1.44] |
| Sex (male) | 1.24 [1.02, 1.51] |
| Age | 0.99 [0.98, 1.00] |
| Retransplantation | 0.69 [0.46, 1.02] |
| ALF | 0.59 [0.39, 0.91]^*^ |
| MELD | 0.98 [0.96, 0.99]^*^ |
| Diabetes | 1.08 [0.86, 1.35] |
| CVP | 0.99 [0.97, 1.00] |
| CIT | 0.99 [0.96, 1.03] |
| Vena cava clamping | 1.00 [0.99, 1.00] |
| Piggyback | 0.59 [0.35, 0.99]^*^ |
| Preoperative creatinine | 1.00 [1.00, 1.00] |
| Preoperative hemoglobin | 1.00 [0.99, 1.00] |

*^*^ Significant at alpha level = 0.05.*

*A HR > 1 increases the risk of discharge. Results are expressed with 95% confidence intervals.*

*This model was a multivariable model stratified for exposure to starch.*

*Interaction between vasopressor and fluid balance was not significant.*

*Abbreviations: ICU = Intensive Care Unit, HR = hazard ratio, ALF = acute liver failure, MELD = Model for End-stage Liver Disease, CVP = central venous pressure, CIT = cold ischemia time*

**Table S5. Results from sensitivity analyses with vasopressor doses as a continuous variable in models without interaction**

| **Outcome** | **Type of relative risk** | **Effect in equivalent of 10 μg per minute of norepinephrine** |
| --- | --- | --- |
| **48-hour AKI** | **POR** | 1.04 [0.94, 1.14] |
| **7-day AKI** | **POR** | 0.95 [0.84, 1.08] |
| **Time to first extubation** | **HR** | 0.85 [0.78, 0.94]^*^ |
| **Time to ICU discharge** | **HR** | 0.92 [0.87, 0.97]^*^ |

*^*^ Significant at alpha level = 0.05.*

*A POR > 1 increases the risk of AKI and a HR > 1 increases the risk of extubation or discharge. Results are expressed with 95% confidence intervals.*

*When interaction was significant, the effect of vasopressor doses was explored in the subgroup of patients with vasopressors (survival model).*

*Abbreviations: AKI = Acute Kidney Injury, ICU = intensive care unit, POR = Proportional Odds Ratio, HR = Hazard Ratio, RRT = renal replacement therapy.*

**Table S6. Results from sensitivity analyses on complete cases only**

1. **AKI**

| **Variables** | **48-hour AKI (POR)**  **(n=416)** | **7-day AKI (POR)**  **(n=410)** |
| --- | --- | --- |
| Any vasopressor upon ICU admission | 1.04 [0.72, 1.50] | 1.21 [0.80, 1.84] |
| Fluid balance (L) | 1.00 [0.95, 1.05] | 1.04 [0.97, 1.11] |
| Intraoperative phlebotomy | 0.85 [0.56, 1.30] | 1.28 [0.77, 2.11] |
| Age (years) | 1.02 [1.01, 1.04]^*^ | 1.02 [0.99, 1.04] |
| Sex (male) | 0.87 [0.58, 1.29] | 1.05 [0.66, 1.68] |
| Retransplantation | 0.65 [0.29, 1.43] | 0.74 [0.32, 1.70] |
| ALF | 0.82 [0.31, 2.19] | 1.69 [0.65, 4.39] |
| MELD | 1.04 [1.01, 1.08]^*^ | 1.03 [0.99, 1.06] |
| Diabetes | 1.50 [0.93, 2.41] | 1.43 [0.85, 2.42] |
| Baseline CVP (mmHg) | 1.01 [0.97, 1.04] | 1.02 [0.98, 1.06] |
| CIT (hours) | 1.11 [1.03, 1.19]^*^ | 1.07 [0.98, 1.17] |
| Vena cava clamping time (minutes) | 1.00 [0.99, 1.02] | 1.01 [0.99, 1.01] |
| Baseline hemoglobin (g/L) | 1.00 [0.99, 1.01] | 0.99 [0.98, 1.01] |
| Baseline creatinine (10 μmol/L) | 0.97 [0.92, 1.02] | 1.03 [0.98, 1.09] |
| Piggyback | 0.56 [0.23, 1.38] | 1.42 [0.57, 3.51] |
| Any intraoperative starch | 1.08 [0.75, 1.55] | 0.90 [0.59, 1.38] |

*^*^ Statistically significant at alpha = 0.05.*

*Results are expressed with 95% confidence intervals.*

*These results come from a multivariable model. Interaction between fluid balance and presence of vasopressor was not significant.*

*Abbreviations: AKI = acute kidney injury, POR = proportional odds ratio, ALF = acute liver failure, MELD = Model for End-stage Liver Disease, CVP = central venous pressure, CIT = cold ischemia time*

1. **RRT**

| **Variable** | **OR (n=417)** |
| --- | --- |
| **Patients without vasopressor upon ICU admission** | |
| Fluid balance (L) | 1.65 [1.15, 2.37]^*^ |
| Intraoperative phlebotomy | 0.68 [0.14, 3.43] |
| **Patients with vasopressors upon ICU admission** | |
| Vasopressor dose^+^ | 1.01 [0.99, 1.02] |
| Fluid balance (L) | 1.02 [0.87, 1.19] |
| Intraoperative phlebotomy | 0.23 [0.09, 3.73] |

^+^ *In equivalent of 10 ug/min of norepinephrine.*

*^*^ Statistically significant at alpha = 0.05.*

*Results are expressed with 95% confidence intervals.*

*P value for interaction = 0.018*

*There were 221 patients with 11 RRT events in the subgroup without vasopressor and 196 patients with 17 RRT events in the subgroup with vasopressors.*

1. **Time to extubation**

| **Variable** | **HR (n=412)** |
| --- | --- |
| Any vasopressor | 0.72 [0.49, 1.04] |
| Fluid balance (L) | 1.00 [0.49, 1.04] |
| Intraoperative phlebotomy | 1.09 [0.80, 1.47] |
| Sex (male) | 1.39 [1.01, 1.89]^*^ |
| Age | 1.02 [1.00, 1.03] |
| Retransplantation | 1.10 [0.59, 2.06] |
| ALF | 0.33 [0.11, 0.97]^*^ |
| MELD | 0.99 [0.97, 1.01] |
| Diabetes | 1.37 [0.98, 1.91] |
| CVP | 0.99 [0.97, 1.02] |
| CIT | 1.03 [0.97, 1.10] |
| Vena cava clamping | 0.99 [0.98, 1.00] |
| Piggyback | 0.70 [0.32, 1.51] |
| Preoperative creatinine | 1.00 [0.99, 1.00] |

1. **Time to ICU discharge**

| **Variable** | **HR (n=417)** |
| --- | --- |
| Any vasopressor | 0.91 [0.62, 1.33] |
| Fluid balance (L) | Non-linear effect^**^ |
| Intraoperative phlebotomy | 1.10 [0.80, 1.51] |
| Sex (male) | 1.37 [1.04, 1.81] |
| Age | 1.00 [0.99, 1.02] |
| Retransplantation | 0.94 [0.50, 1.76] |
| ALF | 0.40 [0.20, 0.83]^*^ |
| MELD | 0.98 [0.96, 1.00] |
| Diabetes | 0.84 [0.59, 1.18] |
| CVP | 1.02 [0.99, 1.04] |
| CIT | 0.98 [0.93, 1.04] |
| Vena cava clamping | 1.00 [0.99, 1.00] |
| Piggyback | 0.61 [0.29, 1.29]^*^ |
| Preoperative creatinine | 1.00 [0.99, 1.00] |
| Preoperative hemoglobin | 0.99 [0.99, 1.00] |

*^*^ Statistically significant at alpha = 0.05*

*^**^ p =0.018*

1. **Survival**

| **Variable** | **HR (n=417)** |
| --- | --- |
| **Patients without vasopressor upon ICU admission** | |
| Fluid balance (L) | 1.71 [1.37, 2.13]^*^ |
| Intraoperative phlebotomy | 0.23 [0.07, 0.86]^*^ |
| **Patients with vasopressors upon ICU admission** | |
| Vasopressor dose^+^ | 1.03 [1.01, 1.05]^*^ |
| Fluid balance (L) | 1.22 [0.97, 1.53] |
| Vasopressor dose*time (month)^++^ | 0.99 [0.99, 1.00] |
| Fluid balance*time (month)^++^ | 0.97 [0.94, 1.00] |

*^+^ In equivalent of 10 ug/min of norepinephrine.*

*^++^ This suggests that the effect weans off over time.*

*^*^ Statistically significant at alpha = 0.05*

*Results are expressed with 95% confidence intervals.*

*p values < 0.001 for interaction between fluid balance and presence or absence of vasopressors (in a model with stratification for vasopressin infusion and exposure to a phlebotomy).*

*There were 221 patients with 13 death events in the subgroup without vasopressor and 196 patients with 15 death events in the subgroup with vasopressors.***Table S7. Exploratory analyses for change of estimates due to confounding from main models**

1. **AKI**

| **Independent variables** | **48-hour KI POR** | | |
| --- | --- | --- | --- |
|  | Unadjusted | Adjusted without vasopressors or fluid balance^++^ | Adjusted for all covariates^+^ |
| Vasopressor (any) | 1.17 [0.86, 1.60] | 1.01 [0.72, 1.41] | 1.01 [0.72, 1.40] |
| Fluid balance (L) | 0.96 [0.92, 1.00] | 0.99 [0.94, 1.04] | 0.99 [0.94, 1.04] |

| **Independent variables** | **7-day AKI POR** | | |
| --- | --- | --- | --- |
|  | Unadjusted | Adjusted without vasopressors or fluid balance^++^ | Adjusted for all covariates^+^ |
| Vasopressor (any) | 1.24 [0.87, 1.77] | 1.09 [0.75, 1.59] | 1.10 [0.76, 1.61] |
| Fluid balance (L) | 1.00 [0.95, 1.06] | 1.04 [0.97, 1.10] | 1.04 [0.97, 1.10] |

1. **RRT (in subgroup with vasopressors)**

| **Independent variables** | **OR** | | |
| --- | --- | --- | --- |
|  | Unadjusted | Adjusted without vasopressors or fluid balance^++^ | Adjusted for all covariates^+^ |
| Fluid balance (L) | 0.98 [0.85, 1.14] | 1.02 [0.85, 1.23] | 1.04 [0.91, 1.17] |

1. **Time to first extubation**

| **Independent variables** | **HR** | | |
| --- | --- | --- | --- |
|  | Unadjusted | Adjusted without vasopressors or fluid balance^++^ | Adjusted for all covariates^+^ |
| Vasopressors (any) | 0.67 [0.56, 0.79]^*^ | 0.64 [0.51, 0.81]^*^ | 0.64 [0.50, 0.81]^*^ |
| Fluid balance (L) | 0.99 [0.97, 1.02] | 1.00 [0.96, 1.04]^++++^ | 0.99 [0.95, 1.04] |

1. **Time to ICU discharge**

| **Independent variables** | **HR** | | |
| --- | --- | --- | --- |
|  | Unadjusted | Adjusted without fluid balance^++^ | Adjusted for all covariates^+^ |
| Vasopressors (any) | 0.83 [0.69, 0.98]^*^ | 0.91 [0.75, 1.09] | 0.91 [0.76, 1.09] |

1. **Survival (subgroup with vasopressors)**

| **Independent variables** | **HR** | | |
| --- | --- | --- | --- |
|  | Unadjusted^+++++^ | Adjusted without vasopressors or fluid balance^++^ | Adjusted for all covariates^+^ |
| Vasopressors (dose)^+++^ | 1.26 [1.13, 1.39]^*^ | 1.29 [1.15, 1.45]^*^ | 1.29 [1.13, 1.49]^*^ |
| Fluid balance (L) | 1.22 [0.99, 1.51] | 1.25 [1.03, 1.51]^*^ | 1.24 [1.01, 1.54]^*^ |

*^*^ Significant at alpha level = 0.05.*

*^+^ As reported in main tables.*

*^++^ Adjusted for all other covariables in the main model.*

*^+++^ In equivalent of 10μg/minute of norepinephrine.*

*^++++^ Has a significant non-linear effect that is non-significant when adjusted for vasopressors.* [4]

*^+++++^ Models were fitted with a coefficient-time interaction.*

*Results are expressed with 95% confidence intervals.*

*N.B. Due to non-linear effect of fluid balance in the time to ICU discharge model, we did not explore estimates modification due to confounding for this association.*

*Abbreviations: AKI = Acute Kidney Injury, POR = Proportional Odds Ratio, RRT = Renal Replacement Therapy, OR = Odds Ratio, HR = Hazard Ratio*

**Table S8. Combined effect measure with interaction on survival**

|  | **Vasopressors – (HR)** | **Vasopressors + (HR)** |
| --- | --- | --- |
| Fluid balance | 1.71 [1.26-2.34] | 3.85 [0.94-15.9] |
| **Other coefficients from the same model** | | |
| Effect of vasopressors when fluid balance = 0 (HR) | 3.47 [0.83, 14.51] | |
| Multiplicative interaction factor (HR) | 0.67 [0.49, 0.92] | |
| **Interaction on an additive scale** | | |
| Relative Excess Risk due to Interaction | -0.28 [-0.91 to 0.35] | |

*From a Cox model stratified for vasopressin infusion and exposure to a phlebotomy (for proportionality reasons) and fitted with a propensity score for exposure to a phlebotomy (preoperative hemoglobin value, preoperative creatinine value, baseline CVP, age, preoperative MELD score and transplantation for acute liver failure).*

*HR = hazard ratio*

Comments on table S8:

There was no significant interaction on the additive scale. Since presence of any vasopressor was not significantly associated with survival in the fitted model (HR = 3.47 [0.83, 14.51]) a significant interaction on only one scale still suggests true mechanistic interaction.[*Knol MJ, VanderWeele TJ. Recommendations for presenting analyses of effect modification and interaction. International Journal of Epidemiology. 2012;41: 514–520*]

**Figure S2. Flowchart**

**Not exclusive categories*

**Figure S3. Survival according to presence of vasopressors upon ICU admission**

**
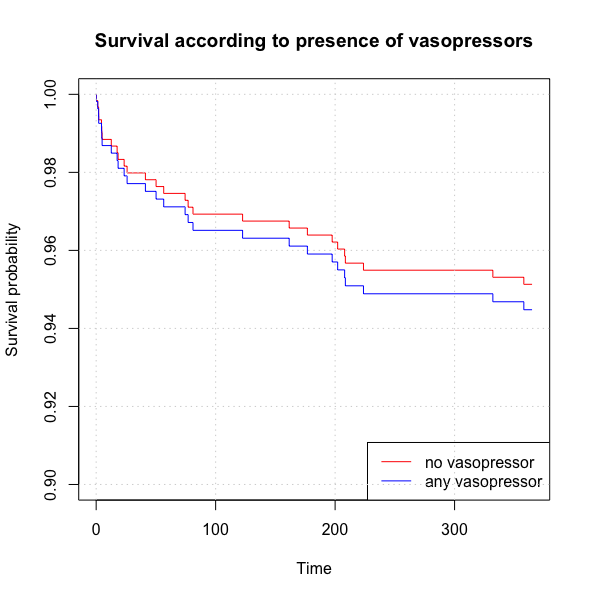
**

*Curves were produced from a Cox model without interaction between fluid balance and vasopressors from complete cases only. Since statistical interaction between vasopressors and fluid balance was significant, these curves are exploratory, and no statistical test is reported. However, vasopressor effect was not significant in an exploratory model without fluid balance as a covariable ((HR = 1.00 [0.41, 2.44]).*

**Figure S4. Fluid-dependent ICU discharge hazard ratio**

**
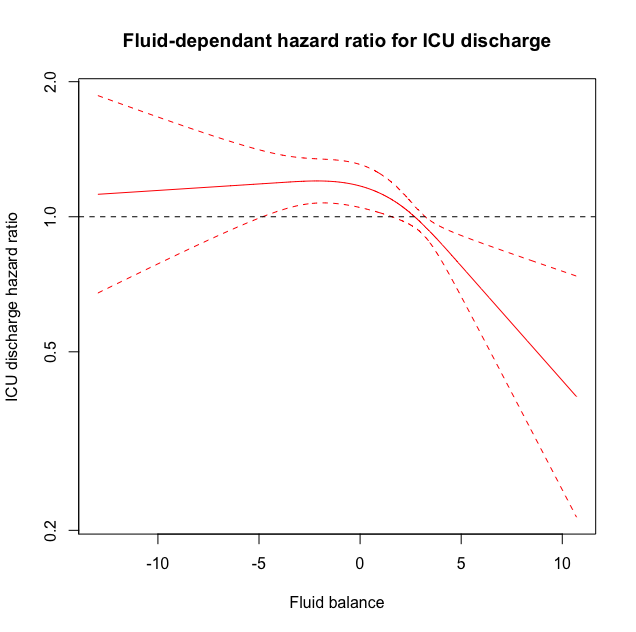
**

*p = 0.001 for effect.*
